# Airway regeneration using iPS cell-derived airway epithelial cells with Cl^-^ channel function

**DOI:** 10.1080/19336950.2019.1628550

**Published:** 2019-06-14

**Authors:** Susumu Yoshie, Koichi Omori, Akihiro Hazama

**Affiliations:** a Department of Cellular and Integrative Physiology, School of Medicine, Fukushima Medical University, Fukushima, Japan; b Department of Otolaryngology Head and Neck Surgery, Graduate School of Medicine, Kyoto University, Kyoto, Japan

**Keywords:** iPS cells, airway epithelial cells, Cl^−^ channel, CFTR, cystic fibrosis

## Abstract

induced pluripotent stem (iPS) cells can be differentiated into various cell types, including airway epithelial cells, since they have the capacity for self-renewal and pluripotency. Thus, airway epithelial cells generated from iPS cells are expected to be potent candidates for use in airway regeneration and the treatment of airway diseases such as cystic fibrosis (CF). Recently, it was reported that iPS cells can be differentiated into airway epithelial cells according to the airway developmental process. These studies demonstrate that airway epithelial cells generated from iPS cells are equivalent to their in vivo counterparts. However, it has not been evaluated in detail whether these cells exhibit physiological functions and are fully mature. Airway epithelial cells adequately control water volume on the airway surface via the function of Cl^−^ channels. Reasonable environments on the airway surface cause ciliary movement with a constant rhythm and maintain airway clearance. Therefore, the generation of functional airway epithelial cells/tissues with Cl^−^ channel function from iPS cells will be indispensable for cell/tissue replacement therapy, the development of a reliable airway disease model, and the treatment of airway disease. This review highlights the generation of functional airway epithelial cells from iPS cells and discusses the remaining challenges to the generation of functional airway epithelial cells for airway regeneration and the treatment of airway disease.

## Introduction

There are many cases in which a central airway, such as the trachea, must be resected due to trauma, inflammation, or thyroid cancer. Although various anastomotic operation methods following airway resection have been reported [1,2], these treatments cause postoperative scarring, and granulation is sometimes observed. Autografts and allografts using costal cartilage, auricular cartilage, and skin have also been performed for airway replacement [3–5]. However, it is difficult to obtain an adequate quantity and appropriate shape of tissue for airway regeneration. Artificial material [6,7] has also been used for central airway regeneration. While this method achieves good clinical outcomes, at least two months are necessary for complete epithelial regeneration on the airway surface of the artificial trachea. The airway epithelium has important functions, such as the regulation of water volume on the airway surface via the transport function of Cl^−^ channels to maintain the mucociliary transport system. Thus, since the regeneration of the airway epithelium must be completed as soon as possible, novel treatment methods for airway regeneration are needed.

Cystic fibrosis (CF) is an autosomal recessive, lethal genetic disease caused by mutations in the *cystic fibrosis membrane conductance regulator* (*CFTR*) gene, which encodes one of Cl^−^ channels. Deficient and/or defective CFTR protein in airway epithelial cells results in decreased Cl^−^ secretion and leads to a reduced water volume on the airway surface, increased viscosity, and impairment of the mucociliary transport system [8]. Therefore, lung damage and respiratory failure are caused by severe airway obstruction, bacterial infections, and chronic inflammation. Ninety percent of CF patients die due to recurrent pulmonary infections. CF disease is the most common hereditary disease in white people of northern Europe and the US, affecting approximately 1 in 2000–4000 newborns [9–11]. More than 2000 mutations in *CFTR* have been reported, and these mutations are divided into seven classes [12–15]. Class I mutations contribute to protein production defects and include nonsense mutations causing degradation of mRNA by nonsense-mediated decay. Class II mutations result in protein processing abnormalities leading to defects in cell surface localization. Class III mutations contribute to dysfunctional channel gating at the apical surface. Class IV mutations affect the reduction of channel conductance. Class V mutations lead to a reduced amount of CFTR protein due to abnormal RNA splicing. Class VI mutations cause protein destabilization at the apical surface due to increased protein turnover. Class VII mutations are so-called unrescuable mutations because of large deletions in the *CFTR* genomic sequence [15,16]. Since there is no curative therapy for CF patients in any class, symptomatic therapies involving a pharmacological approach have mainly been adopted, and effective therapies are still in the research stage. Several studies using *CFTR* knockout mice to test available treatments have been reported [17–19]. However, these mice do not display the CF disease-associated phenotype observed in human CF disease. Thus, a reliable CF disease model showing a phenotype similar to that of human CF disease must be constructed.

**Figure 1.**
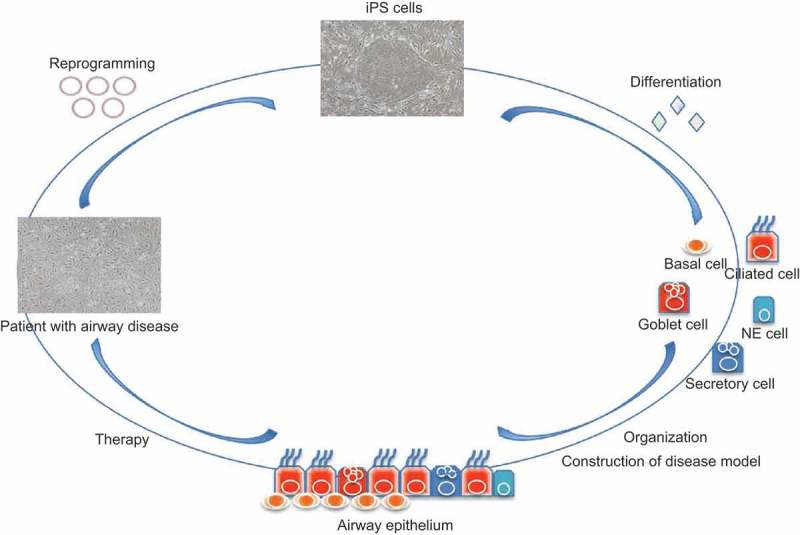
Schema of the application process for airway regeneration using iPS cell technology. iPS cells are generated from patient somatic cells by overexpression of reprogramming factors. Functional airway epithelial cells (ciliated, goblet, basal, secretory, and NE cells) are induced from iPS cells. Construction of the patterned airway epithelium and disease model is performed for airway regeneration and the treatment of airway diseases such as CF.

Embryonic stem (ES) cells that are generated from the inner cell mass of blastocyst-stage embryos exhibit self-renewal and pluripotency abilities [20,21]. They can give rise to cells of all three germ layers and many different cell types under appropriate conditions, and they have been frequently suggested as a potential cell source for regenerative therapy. However, the establishment of ES cells requires the destruction of preimplantation embryos at the blastocyst stage, which is highly morally contentious. Moreover, the transplantation of ES cells for therapeutic purposes triggers host immune rejection. In 2006 and 2007, induced pluripotent stem (iPS) cells established from somatic cells by overexpression of reprogramming factors were shown to present self-renewal and pluripotency abilities similar to those of ES cells [22,23]. These cells can be induced to become various cell types with a specific function under appropriate conditions. The use of iPS cells has given rise to new possibilities for regenerative therapy based on cell/tissue transplantation as well as research on various diseases, as there have been issues of immune system rejection and ethical controversy with regard to the use of ES cells. Thus, functional airway epithelial cells derived from iPS cells are expected to be a useful cell source for airway regeneration and the treatment of airway disease (Figure 1). Several research groups have reported the generation of airway epithelial cells from iPS cells [24–35]. Here, we review recent progress focused on the generation of iPS cell-derived airway epithelial cells with physiological functions and discuss the remaining challenges to the generation of functional airway epithelial cells.

## The various specialized cells in the airway epithelium

The upper and central airway epithelium are composed of ciliated cells, goblet cells, and basal cells. In particular, ciliated cells are the predominant cell type within the airway, accounting for over 50% of all airway epithelial cells, and these cells control the water volume on the airway surface via the transport function of Cl^−^ channels and perform directional transport of inhaled particles via ciliary movement [36,37]. Goblet cells produce mucus to trap foreign objects [38,39], and basal cells are thought to be heterogeneous stem cell populations giving rise to ciliated cells and goblet cells [40–43]. In the distal bronchus and bronchioles, secretory cells such as Clara cells are abundant, and a small number of neuroendocrine (NE) cells are also present [44]. Secretory cells produce bronchiolar surfactant to prevent the harmful effects of the inhalation of, for example, foreign substances and carcinogens, and they serve as progenitors for both ciliated and goblet cells [45]. NE cells are thought to function as chemoreceptors and as a component of the stem cell niche, in addition to being the cells of origin in small-cell lung cancer [46,47]. However, the detailed functions of secretory cells and NE cells remain unclear, and unidentified cells may be present in the airway epithelium.

## Functional evaluation of airway epithelial cells from iPS cells

There are several reports of the differentiation of airway epithelial cells from human/mouse iPS cells by using a stepwise developmentally guided strategy (Table 1). Green et al (2011). have succeeded in the generation of airway epithelial cells from iPS cell-derived anterior foregut endoderm using an in vivo microenvironment or niche. Mou et al (2012). have induced the differentiation of iPS cells into cells positive for Nkx2.1- and Sox2, which are indicators of airway progenitor cells, by the precisely timed addition of BMP, FGF, and Wnt. However, these reports have not included airway epithelial cell-specific functional analysis. Wong et al (2012). demonstrated the differentiation of airway epithelial cells from CF patient iPS cells, examined the maturity of the CFTR protein by immunostaining and western blot analysis, and analyzed CFTR channel activity using a halide efflux assay. Furthermore, the measurement of CFTR currents by whole-cell patch clamp experiments has also been performed by Firth et al (2014 and 2015). Additionally, Crane et al (2015). and Suzuki et al (2016). have measured the short circuit current of CFTR-dependent Cl currents by Ussing chamber analysis, and McCauley et al (2017). have reported forskolin-induced CFTR-dependent organoid swelling. On the other hand, Konishi et al (2016). examined airway epithelial cell-specific ciliary movement with a constant rhythmic rate using a high-speed camera. Our group has also investigated ciliary movement with constant rhythm using a high-speed camera and the transport function of CFTR using a halide ion sensor [35]. In all of these reports, the analyses of gene expression, proteins, and morphology indicate that the characteristics of iPS cell-derived airway epithelial cells are similar to those of their in vivo counterparts. However, there are few reports comparing the functions of iPS cell-derived airway epithelial cells with those of native airway epithelial cells. It remains unknown whether iPS cell-derived airway epithelial cells are immature or mature. A transplantation experiment using iPS cell-derived airway epithelial cells has also been reported for airway regeneration [48]. While the survival of iPS cell-derived airway epithelial cells was confirmed at the site of a defect or injury in immunodeficient mice, functional assays, such as those examining the transport function of Cl^−^ channels to maintain the mucociliary transport system, were not performed. Hence, neither in vitro nor in vivo studies have yet included detailed evaluations of whether iPS cell-derived airway epithelial cells exhibit physiological functions such as the transport function of Cl^−^ channels and whether these cells are fully mature.

**Table 1.** Functional assessment of iPS cell-derived airway epithelial cells.

Author (Year)	Functional assessment	Function
Green *et a*l. (2011)	−	−
Mou *et al*. (2012)	−	−
Wong *et al*. (2012)	+	CFTR
Huang *et al*. (2014)	−	−
Firth *et al*. (2014 and 2015)	+	CFTR
Dye *et al*. (2015) Crane *et al*. (2015) Konishi *et al*. (2016) Suzuki *et al*. (2016) McCauley *et al*. (2017) Yoshie *et al*. (2019)	− + + + + +	− CFTR Ciliary Movement CFTR CFTR, Ciliary Movement CFTR, Ciliary Movement

## Function of Cl^−^ channels on the airway epithelium

**Figure 2.**
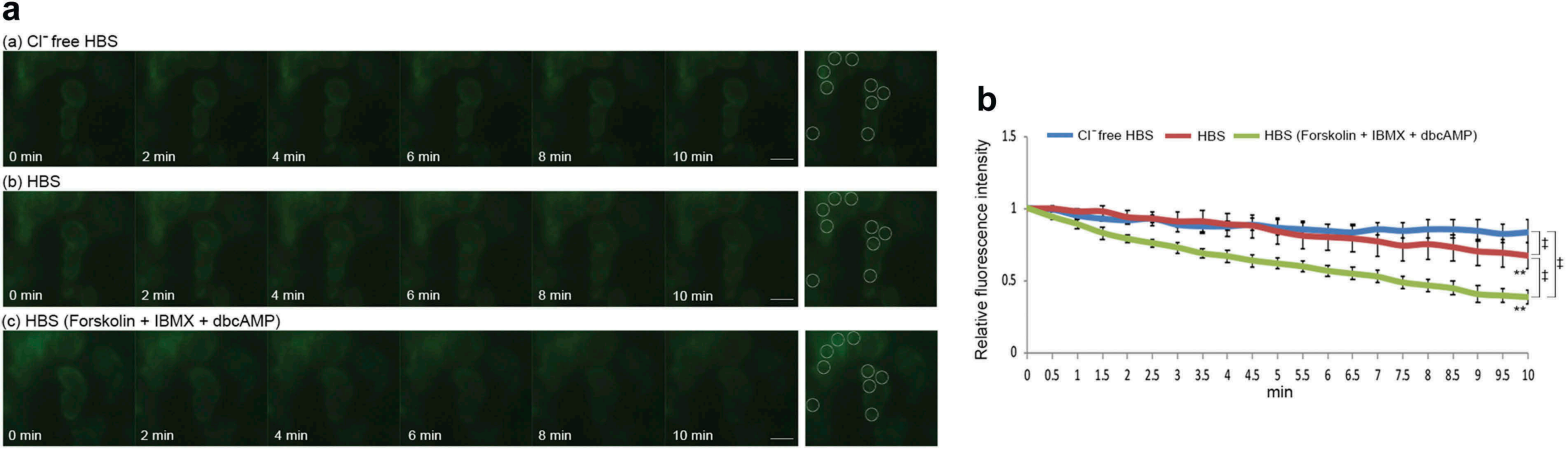
Transport function of the Cl^−^ channels and CFTR in iPS cell-derived airway epithelial cells. (a) Time-lapse fluorescent images of mYFP-labeled iPS cell-derived airway epithelial cells in Cl^–^free HBS (a), normal HBS (b), and normal HBS plus 10 μM Forskolin, 100 μM IBMX and 1 mM dbcAMP (c). (b) Time-dependent relative fluorescence intensity of regions of interest (ROIs) in mYFP-labeled iPS cell-derived airway epithelial cells in various HBS conditions. The fluorescence intensity at each time point was compared with that at 0 min. **P < 0.01 vs time 0; ‡*P* < 0.01.

The airway epithelium exhibits important functions such as the regulation of mucus volume and water volume on the airway surface to maintain mucociliary transport system function. The luminal side of airway epithelium is covered with airway surface liquid (ASL), which is composed of a mucus layer that traps inhaled particles from the external environment, and a periciliary liquid layer (PCL), which is a water layer that maintains a reasonable distance between the mucus and epithelium [49,50]. The regulation of PCL volume is indispensable for the maintenance of the mucociliary transport system, and the transfer of water molecules to the luminal side mainly depends on Cl^−^ transport via Cl^−^ channels [51]. When Cl^−^ is secreted to the luminal side via CFTR and/or other Cl^−^ channels, such as TMEM16A, which is a calcium-activated Cl^−^ channel, the electric potential on the luminal side temporarily shifts to a negative charge. The electrochemical gradient due to Cl^−^ secretion promotes Na^+^ transport to the luminal side, and the resultant osmotic pressure difference between the luminal and serosal sides causes the transport of water molecules to the luminal side. Conversely, when Na^+^ and Cl^−^ are absorbed into the cell via epithelial Na^+^ channels (ENaC) and Cl^−^ channels, the absorption of water molecules is promoted, and the PCL volume is decreased. In particular, since CF patients exhibit dysfunction of CFTR, Cl^−^ secretion to the luminal side is decreased compared to that in healthy patients. Furthermore, ENaC activation by CFTR dysfunction causes Na^+^ absorption from the luminal side, and the resultant environment increases viscosity on the airway surface [52–54]. Thus, CFTR and other Cl^−^ channels have an important function in maintaining the mucociliary transport system on the airway surface. We evaluated the transport function of Cl^−^ channels in iPS cell-derived airway epithelial cells with mutant YFP (mYFP) containing two point mutations at position 148 (histidine) and 163 (valine), which shows high sensitivity to halide ions. The absorption spectrum of mYFP modified with halide ions is decreased, and its fluorescence is quenched [55–57]. Hence, following the fluorescence intensity of mYFP can indicate the transport function of Cl^−^ channels in iPS cell-derived airway epithelial cells. The extracellular fluid of iPS cell-derived airway epithelial cells was replaced with Cl^–^free HBS from differentiation medium. After 1 hr, Cl^–^free HBS was replaced with HBS or HBS including a cocktail (10 μM Forskolin, 100 μM IBMX, and 1 mM dbcAMP) that can activate CFTR. When iPS cell-derived airway epithelial cells were continuously incubated with Cl^–^free HBS, there was no notable change in fluorescence intensity (Figure 2a). On the other hand, when Cl^–^free HBS was replaced with HBS, the fluorescence intensity was decreased in a time-dependent manner (Figure 2b), and a significant difference was confirmed between 0 min and 10 min after buffer replacement. Furthermore, when iPS cell-derived airway epithelial cells were incubated with HBS including the cocktail, the fluorescence intensity was immediately decreased (Figure 2c). A significant difference in the fluorescence intensity was shown between cells treated with Cl^–^free HBS, HBS, and HBS with the cocktail after 10 min of medium replacement (Figure 2b). The data presented here indicate that CFTR and other Cl^−^ channels in iPS cell-derived airway epithelial cells transported Cl^−^ into the cells according to the concentration gradient of Cl^−^. Since these results were similar to those of a previous report [35], this assay is considered to be an effective method for evaluating the transport function of Cl^−^ channels.

## Establishment of iPS cells from CF patients

iPS cells are effective tools not only for use in regenerative medicine, such as cell/tissue replacement, but also as a disease model. *CFTR* knockout mice cannot mimic the human-specific CF phenotype due to species differences. Thus, the establishment and use of CF-specific human iPS cells is expected to provide a powerful tool for the treatment of CF. The most common cause of CF is the deletion of phenylalanine 508 in *CFTR*. The deletion of phenylalanine at residue 508 in the amino acid sequence of the protein occurs in at least 1 allele in approximately 90% of CF patients [58]. This mutation causes misfolding, misassembly, mistrafficking, and dysfunction of the CFTR protein [59–61]. For CF patients with this mutation, the dual combination of a CFTR corrector, which improves the transport of protein to the cell surface, and a CFTR potentiator, which increases in the time in which the channel is in the open state, results in an increase in Phe*CFTR*del protein activity and is more effective than either treatment alone [62]. Furthermore, it has been reported that treatments involving triple combinations, such as two correctors and a potentiator, result in increased CFTR function [63,64]. Recently, the establishment of iPS cells from CF patients carrying Phe*CFTR*del has been reported to facilitate the discovery of new mutation-targeted therapies. Wong et al (2012)., Firth et al (2015)., Crane et al (2015)., Suzuki et al (2016)., and Fleischer et al. [65]. established CF iPS cells with Phe*CFTR*del and confirmed defects in the normal expression pattern, plasma membrane localization, and CFTR activity. Moreover, since they indicated that functional recovery of CFTR was observed following the use of a CFTR corrector and genome editing, patient-derived iPS cell-derived airway epithelial cells have been shown to recapitulate the phenotypes of CF in vitro. However, it remains unknown whether these cells were mature airway epithelial cells with clonal properties.

## Future directions

Previous studies have suggested that iPS cells can be differentiated into airway epithelial cells [24–35], but it has not been clarified in detail whether these cells exhibit physiological functions such as the transport function of Cl^−^ channels and are mature. Thus, in addition to the evaluation of iPS cell-derived airway epithelial cells by analyses of gene expression, proteins, and morphology, functional analysis is also indispensable, and the establishment of a functional assay system is essential for verification. Furthermore, since it is difficult to induce iPS cells to differentiate into mature airway epithelial cells with clonal properties, it is necessary to investigate the mechanism of terminal differentiation and search for reliable maturation markers. Hence, single-cell analysis might provide valuable information for the functional assessment of iPS cell-derived airway epithelial cells. The method for evaluation of the transport function of Cl^−^ channels using mYFP described in this review can be analyzed at the single-cell level, so it is considered a powerful assay. The airway epithelium is mainly composed of ciliated cells, and functional analysis of these cells has been performed. On the other hand, goblet cells, secretory cells, and NE cells also exhibit important functions, such as mucus production, surfactant secretion, and chemoreceptor functions. Thus, the function of the cells differentiated from iPS cells must be evaluated. However, in the airway epithelium in vivo, the number of secretory cells and NE cells is a small compared to that of ciliated cells, and their detailed roles have not been clarified. Their functions must be further elucidated for airway regeneration to be achieved. Additionally, the construction of a patterned functional airway epithelium with polarity will be required for transplantation because all specialized cells forming the airway epithelium are regularly aligned on the basement membrane and must exhibit appropriate functions in the airway epithelium. Furthermore, after transplantation, not only must survival assessment be performed, but physiological functions such as Cl^−^ channel function and ciliary movement must also be analyzed.

Therapeutic agents for the treatment of CF disease have been developed using primary airway epithelial cells. However, it is difficult to obtain an adequate quantity of cells and maintain them in culture for an extended period. Furthermore, genetic single nucleotide polymorphisms (SNPs) are partly responsible for the differences in drug effects among individuals. Thus, individual CF patient-derived iPS cells would be an effective tool for the discovery of new mutation-targeted therapies.
